# Technical variability of cornea parameters derived from anterior segment OCT fitted with Fringe Zernike polynomials

**DOI:** 10.1007/s00417-023-06186-y

**Published:** 2023-08-02

**Authors:** Achim Langenbucher, Nóra Szentmáry, Alan Cayless, Jascha Wendelstein, Peter Hoffmann

**Affiliations:** 1 Department of Experimental Ophthalmology, Saarland University, Kirrberger Str 100 Bldg. 22, 66424 Homburg/Saar, Germany; 2 Dr. Rolf M. Schwiete Center for Limbal Stem Cell and Aniridia Research, Saarland University, /Saar, Homburg, Germany; 3 Department of Ophthalmology, Semmelweis-University, Budapest, Hungary; 4 School of Physical Sciences, The Open University, Milton Keynes, UK; 5 Department of Ophthalmology, Johannes Kepler University Linz, Linz, Austria; 6 Augen- und Laserklinik Castrop-Rauxel, Castrop-Rauxel, Germany

**Keywords:** Bootstrap techniques, Anterior segment tomographer, Model parameter uncertainties, Robustness of surface fit, Fringe Zernike polynomial surface representation

## Abstract

**Background:**

This study uses bootstrapping to evaluate the technical variability (in terms of model parameter variation) of Zernike corneal surface fit parameters based on Casia2 biometric data.

**Methods:**

Using a dataset containing *N* = 6953 Casia2 biometric measurements from a cataractous population, a Fringe Zernike polynomial surface of radial degree 10 (36 components) was fitted to the height data. The fit error (height – reconstruction) was bootstrapped 100 times after normalisation. After reversal of normalisation, the bootstrapped fit errors were added to the reconstructed height, and characteristic surface parameters (flat/steep axis, radii, and asphericities in both axes) extracted. The median parameters refer to a robust surface representation for later estimates of elevation, whereas the SD of the 100 bootstraps refers to the variability of the surface fit.

**Results:**

Bootstrapping gave median radius and asphericity values of 7.74/7.68 mm and −0.20/−0.24 for the corneal front surface in the flat/steep meridian and 6.52/6.37 mm and −0.22/−0.31 for the corneal back surface. The respective SD values for the 100 bootstraps were 0.0032/0.0028 mm and 0.0093/0.0082 for the front and 0.0126/0.0115 mm and 0.0366/0.0312 for the back surface. The uncertainties for the back surface are systematically larger as compared to the uncertainties of the front surface.

**Conclusion:**

As measured with the Casia2 tomographer, the fit parameters for the corneal back surface exhibit a larger degree of variability compared with those for the front surface. Further studies are needed to show whether these uncertainties are representative for the situation where actual repeat measurements are possible.


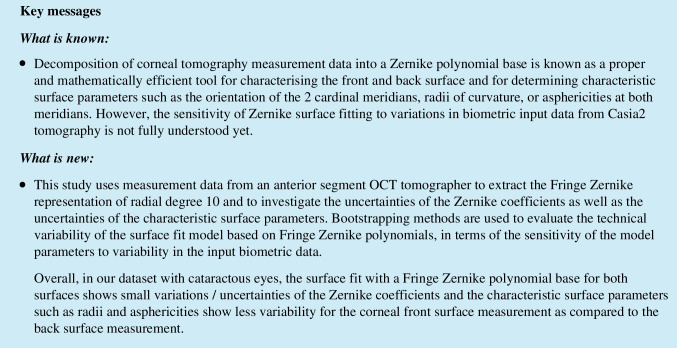


## Introduction

Corneal parameters and surface models for the cornea are mandatory in ophthalmology for special applications such as raytracing through the eye, contact lens fitting, calculation of optical implants including capsular bag replacement lenses (for cataract surgery or clear lens extraction) or supplementary lenses (phakic lenses or Add-On lenses), and for definition of ablation profiles in refractive laser surgery.

There are currently several competing tomography techniques for measuring the corneal front and back surface: in addition to classical Scheimpflug tomography, which uses projections onto the cornea of a thin light slit comparable to a slitlamp biomicroscope, the new generation of anterior segment optical coherence tomographers (OCT) has gained in popularity. The Scheimpflug tomographer uses multiple projections from a rotating or scanning slit to examine the entire cornea, whereas in OCT devices, multiple A-scans are used in a polar grid (multiple meridians) or a raster scan (parallel line scans) to examine the entire cornea. The first generation of time domain OCTs had the disadvantage of a slow scanning rate (e.g. 2000 A-scans per second with the Zeiss Visante), whereas newer generation spectral domain OCTs or swept source OCTs employ scanning rates of up to 100,000 A-scans per second. This allows for a dense sampling (high lateral resolution) together with a short examination time (to avoid movement artifacts).

All tomographers process the measurement data internally in order to fit surface models of varying degrees of complexity to both corneal surfaces [1, 2]. Various clinically relevant parameters such as the average central corneal radius, the radius in both cardinal meridians, together with the orientation axes or the asphericity of the surface can be extracted from these surface models [3]. Additional parameters from a more detailed analysis give information about surface asymmetry, local irregularity, or similarity to an ectatic disease configuration (e.g. keratoconus screening). However, the internal processing of the measurement data is not disclosed.

Nearly all tomographers provide an interface allowing the raw data to be exported [4]. These interfaces provide data on axial or instantaneous curvature or power, keratometric power, surface sag (height data), or elevation (height data minus a reference surface). The reference surface for derivation of the elevation data may be either a simple best fit sphere (floating or centred), or more complex surface models such as quadric or biconic surfaces.

Ophthalmologists are familiar with surface models characterising corneal shape. Best fit spheres derived from measurement data in a central region of the cornea (e.g. a 9-mm zone) are typically very robust, but are restricted to the overall base curve of the cornea and do not fully reflect the imaging properties. In contrast, if the fitting is restricted to smaller zones (e.g. measurement data over the entrance pupil), more complex models (quadric surfaces [2], or a biconic surface [4]), then the alignment of the surface with respect to the instrument axis must be taken into account, and the larger number of degrees of freedom in the surface fit might negatively impact the robustness of the surface fit. In the worst case (e.g. if a biconic or Zernike polynomial surface is fitted to the measurement data), 6 degrees of freedom have to be considered (3 translation and 3 rotation parameters). If, e.g. a decentration of the measurement axis to the centre of the model surface is ignored, a simple spherical aberration component converts to spherical aberration overlaid with tilt and coma (both of which grow in a linear fashion with decentration), and astigmatism and defocus (both of which grow quadratically with decentration) [5].

Surface fits using Zernike polynomials generally have the advantage of being additive [3, 6]. Such polynomials are well known to be orthogonal within the unit circle; however, if we are restricted to discrete data points extracted from the tomographer, they are no longer orthogonal [5]. However, in most cases, an orthogonalisation (e.g. Gram-Schmidt-orthogonalisation) is not required for clinical applications since the correlation of the Zernike polynomials on our data samples is very low. This additive behaviour means that the coefficients of the surface decomposition into Zernike polynomials can easily be derived by solving a least squares equation system [3, 5]. Given the selection of an appropriate radial order for the polynomial base [7, 8], corneal surfaces can be characterised sufficiently with Zernike polynomials. This allows all relevant parameters such as average corneal radius, the radii of curvature in both cardinal meridians together with the orientation axes, as well as the asphericity (or conic constant) overall or in both meridians to be extracted easily [9]. However, it is important to understand whether our surface fit with Zernike polynomials is robust enough to reliably extract these characteristic surface parameters [6, 7, 10]. In most cases, an expansion into a full Zernike polynomial base is not required for clinical applications. With higher radial degrees, the number of azimuthal frequencies increases, and as an example, for a radial degree of *N*_*Z*_ = 4 or 6, a total of 15 or 28 Zernike polynomials have to be considered. Since Zernike polynomials with higher azimuthal frequencies might be mostly affected by noise from the tomographer, in most applications, it is sufficient to consider a simplified polynomial base (the so-called Fringe Zernike polynomials) where the ‘wings’ of the Zernike scheme are clipped in such a way that the sum of the radial degree and the azimuthal order equals *N*_*Z*_. For example, for a radial degree of *N*_*Z*_ = 4 or 6, a total of 9 or 16 Fringe Zernike polynomials have to be considered [5].

The purpose of this study was:

- to better understand the sensitivity of Fringe Zernike corneal surface fitting polynomials with radial degree up to *N*_*Z*_ = 10 to variations in Casia2 anterior segment tomography input data (corneal front and back surface), using bootstrapping of the surface fit error,
- to investigate the technical variability of the surface fit for raytracing applications and to extract characteristic surface parameters such as radii, orientation axes, and asphericities in both cardinal meridians as used in intraocular lens power calculations, and
- to apply this procedure to a large clinical dataset to show the clinical applicability.

## Materials and methods

### Dataset for formula constant optimisation

**Fig. 1:**
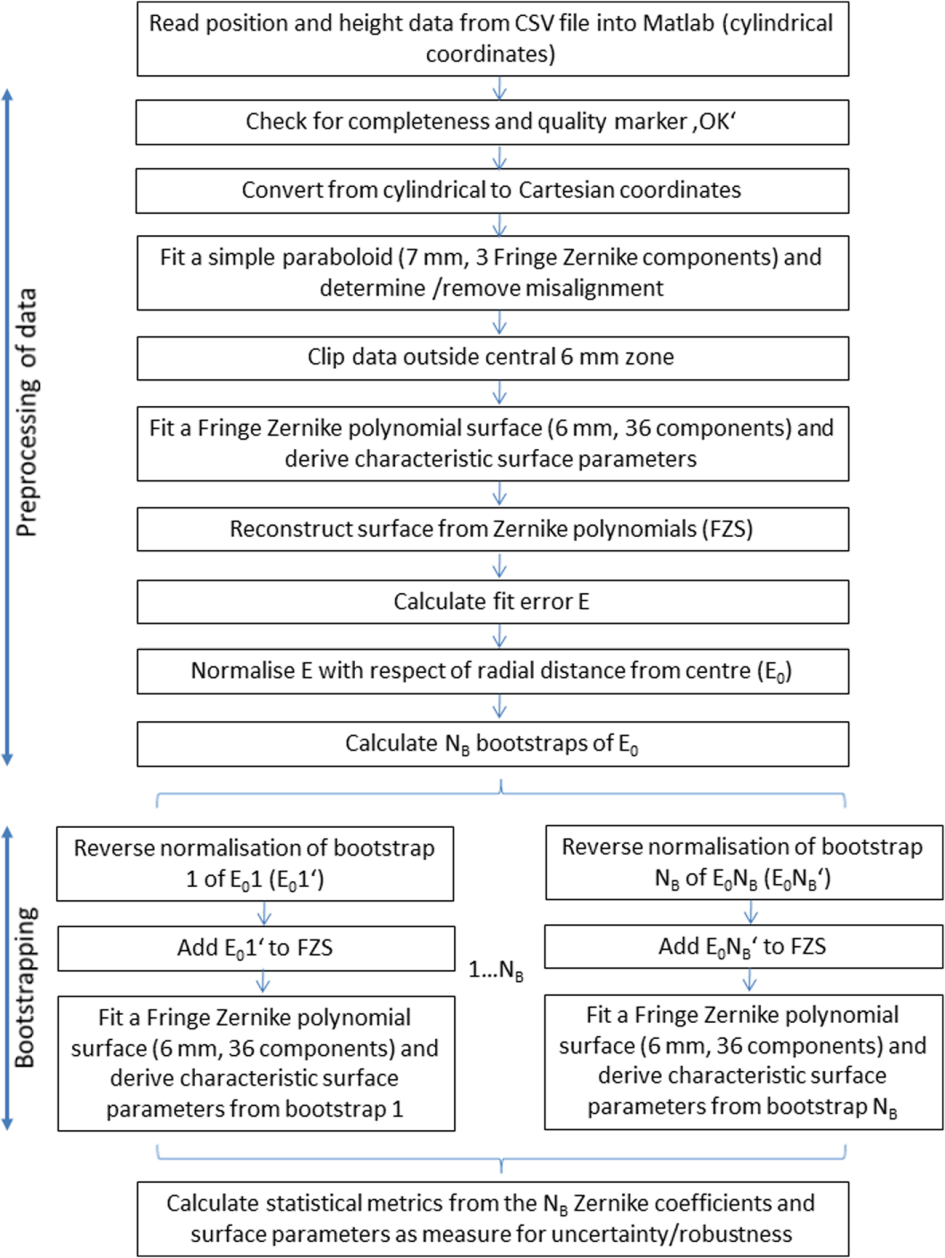
This scheme describes the strategy of surface data analysis. After alignment of the corneal surface, the initial Fringe Zernike decomposition of radial degree *N*_*Z*_ = 12 is performed and the surface reconstruction FZS and the fit error *E* are derived. After normalisation of *E*, *N*_*B*_ bootstraps are calculated. After reverse normalisation, the *N*_*B*_ bootstrapped fit errors are added to FZS, and each bootstrap is decomposed into a Fringe Zernike surface with radial degree *N*_*Z*_ = 12. The metrics for uncertainty or robustness are derived from the *N*_*B*_ bootstrapped Zernike surface representations

In this retrospective study, we analysed a dataset containing measurements from 8000 eyes from a cataract population from the Augen- und Laserklinik Castrop-Rauxel, Castrop-Rauxel, Germany, which was transferred to us. The local ethics committee (Ärztekammer des Saarlandes) provided a waiver for this study (157/21). The raw export data (.CSV-format) were transferred to us in an anonymised fashion, precluding back-tracing of the patient. The anonymised data contained preoperative anterior segment tomographic data acquired using the Casia2 (Tomey GmbH, Nürnberg, Germany, software version Ver.50.5A.03). The CSV data were imported into MATLAB (Matlab 2021a, MathWorks, Natick, USA) for further processing. Figure 1 shows the strategy of data analysis as described below. This strategy is applied independently to the corneal front and corneal back surface data, which are indexed with ()_*a*_ and ()_*p*_.

### Preprocessing of the data

Custom software was written in Matlab. As standard, data exported from the Casia2 software include lateral position data and data on axial, keratometric, or instantaneous curvature/power of both surfaces or real/refractive power of the cornea, as well as height and elevation data. In addition to eye side (OS or OD), gender, date of birth, and examination date, we restricted the data selection to the lateral position and height of the corneal front and back surface, discarding all other data. Each data block (lateral position and surface height of both surfaces) contained cylinder coordinate measurements at 32 semi-meridians (angle *θ* from 0° to 348.75° in steps of 11.25°) with 400 radial positions (radial distance *r* from centre from 0.02 to 8.0 mm in steps of 0.02 mm) each in a central 16-mm zone. Measurements having a quality marker QS other than ‘OK’, and incomplete datasets for the corneal front or back surface height within the 7-mm central zone of the cornea were excluded from the study. The patient age was derived from the examination date and the date of birth.

Cylindrical coordinates (*r*,*θ*,*Z*) were converted to Cartesian coordinates (*X*,*Y*,*Z*) for further processing. Fringe Zernike polynomials were used as shown in Gross [5]. For misalignment removal in the data, a rigid transformation was implemented according to Schröder [4]. A simple ‘floating’ rotational symmetric 4th-order paraboloid (Fringe Zernike polynomials: piston *Z*1, defocus *Z*4, and spherical aberration *Z*9) was fitted to the measurement data within the central 7-mm zone to derive the lateral translation offset and the tilt of the surface. The lateral position data and surface height data for the corneal front and back surface were both corrected for lateral translations (in the *X* and *Y* directions) and tilt (rotation around the *X* and *Y* axes) before further processing. After misalignment removal, all data outside the central 6-mm zone were discarded [4].

Subsequently, an initial Fringe Zernike polynomial surface of degree *N*_*Z*_ = 10 (FZS, in total a Zernike polynomial base with 36 components, with ordering according to Gross [5]) was fitted to the corneal front and back surface height data in terms of a least squares solution from a matrix calculus (unit circle refers to a 6-mm zone). The axes of the two cardinal meridians (flat meridian *A*1 and steep meridian *A*2) were extracted from the Zernike coefficients characterising the primary astigmatism. The FZS reconstruction was analysed at both cardinal meridians *A*1 and *A*2 with a polynomial expansion of radial degree 4 to determine the central radii of curvature (radius *R*1 and *R*2) as well as the asphericities *Q*1 and *Q*2 at the flat and steep meridian, respectively. In addition to *R*1, *A*1, *Q*1 and *R*2, *A*2, *Q*2, the average radius (*R* = 0.5·(*R*1+*R*2)) and asphericity (*Q* = 0.5·(*Q*1+*Q*2)), the differences in radius (Δ*R* = *R*1−*R*2) and asphericity (Δ*Q* = *Q*1−*Q*2), and the projections of Δ*R* to the 0°/90° meridian (*R*0° = Δ*R*·cos(2*A*1)) and the 45°/135° meridian (*R*45° = Δ*R*·sin(2*A*1)) were recorded.

The fit error *E*, defined as the height difference between the measurement data and Fringe Zernike reconstruction, was derived for both surfaces. As the measurement error of anterior segment OCT is known to increase in a quadratic fashion from centre to periphery (*R* = (*X*^2^+*Y*^2^)^1/2^), the absolute value of the error in radial direction was fitted by a 2nd-order polynomial to derive the intercept *a*_0_ and 2nd-order coefficient *a*_2_. The fit error *E* was normalised to:

$${E}_0\left(R,\alpha \right)=\frac{E\left(R,\alpha \right)}{a_0+{a}_2\bullet {R}^2}.$$

### Bootstrapping implementation

The following section outlines the strategy of bootstrapping for estimation of standard errors of the formula constants. This was performed separately for the corneal front and back surfaces.

1. The normalised fit error *E*_0_ was sampled *N*_*B*_ times (*N*_*B*_ refers to the number of bootstrap sequences) with replacement (*E*_0_1 to *E*_0_*N*_*B*_).
2. The normalisation of the *N*_*B*_ bootstrapped fit errors *E*_0_1 was reversed (*E*_0_*N*_*B*_) by
  $${\displaystyle \begin{array}{ccc}{E}_0{1}^{\prime}\left(R,\alpha \right)& =& {E}_01\left(R,\alpha \right)\bullet \left({a}_0+{a}_2\bullet {R}^2\right)\\ {}\dots & \dots & \dots \\ {}{E}_0{N_B}^{\prime}\left(R,\alpha \right)& =& {E}_0{N}_B\left(R,\alpha \right)\bullet \left({a}_0+{a}_2\bullet {R}^2\right)\end{array}}.$$

3. The bootstrap errors after reversion *E*_0_1’ to *E*_0_*N*_*B*_’ were added to the Fringe Zernike surface reconstruction data FZS ([10]). For each bootstrap, a new Fringe Zernike polynomial decomposition of radial degree *N*_*Z*_ = 10 was performed to obtain the 36 Zernike coefficients as described before.
4. The mean, median, SD, and 90% confidence intervals were calculated from the *N*_*B*_ sets of Zernike coefficients. The 90% confidence interval for the *N*_*B*_ sets of Zernike coefficients was then quoted as the ‘uncertainty’ of the Zernike coefficients [11].
5. For each of the *N*_*B*_ bootstraps, the characteristic surface parameters *R*1, *R*2, *R*, Δ*R*, *R*0°, *R*45° and *Q*1, *Q*2, *Q*, Δ*Q* (calculated as shown above) were calculated, and their 90% confidence intervals quoted as the ‘uncertainty’ of the surface characteristics.

### Statistical evaluation

The explorative data for both corneal surfaces are shown with mean, standard deviation (SD), median, and 90% confidence intervals (5% quantile as the lower bound and 95% quantile as the upper bound) for the Fringe Zernike coefficients of the initial surface fit (restricted to the first 9 components referring to the 4th radial degree), the polynomial coefficients *a*_0_ and *a*_2_ describing the radial polynomial fit of the absolute fit error, as well as the standard deviation of the *N*_*B*_ bootstrapped surface characteristics parameters *R*1, *R*2, *R*, Δ*R*, *R*0°, *R*45° and *Q*1, *Q*2, *Q*, Δ*Q*. For the *N*_*B*_ bootstrapped Fringe Zernike coefficients (restricted to the first 9 components referring to the 4th radial degree), the 90% confidence interval is shown.

## Results

From the *N* = 8000 data from the Casia2 tomographer transferred to us, a total of *N* = 6953 (3744 right eyes and 3209 left eyes) were used after eliminating measurements with incomplete data or with a quality check other than ‘OK’. The mean age at the time of measurement was 71.04±13.05 years.

**Fig. 2:**
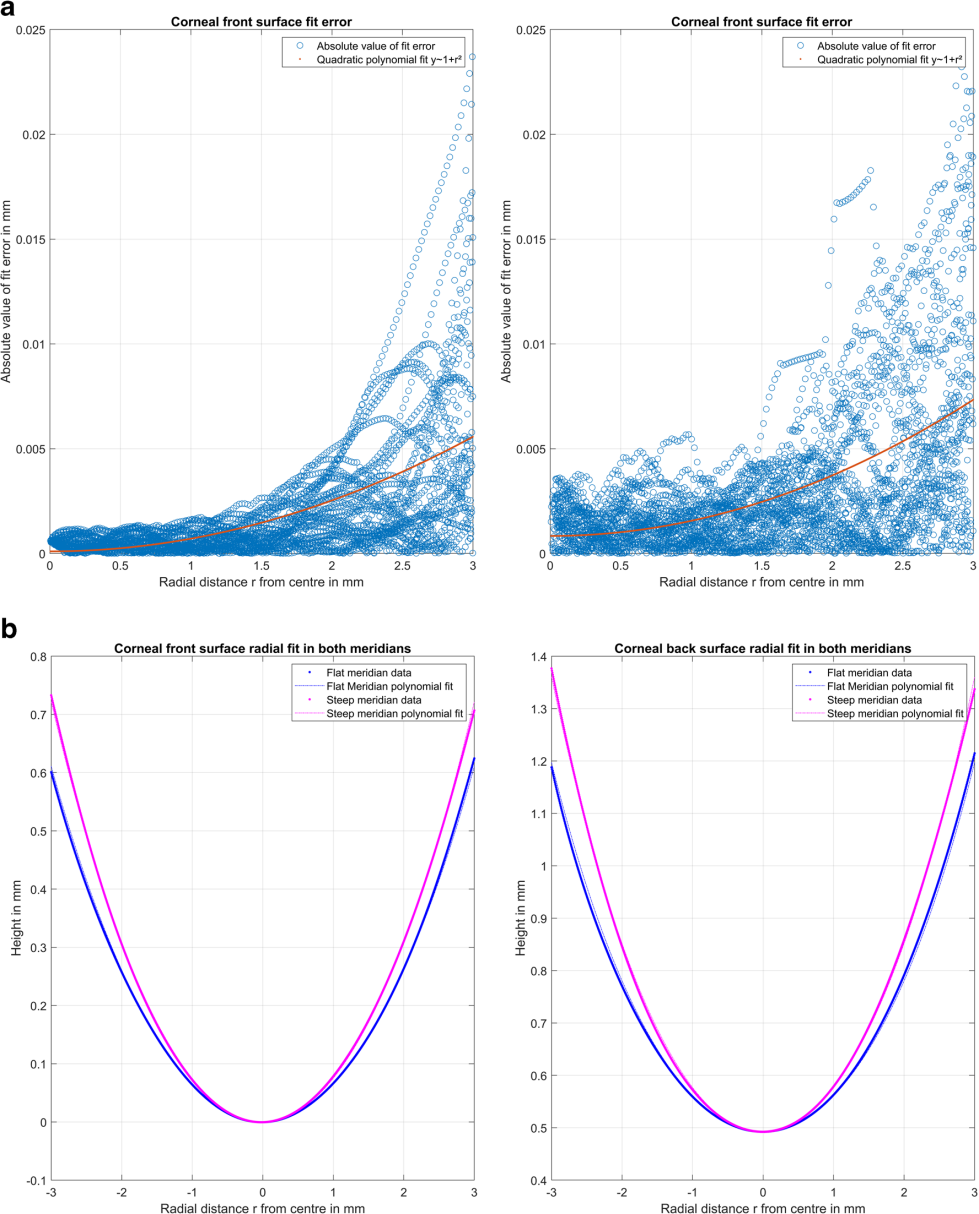

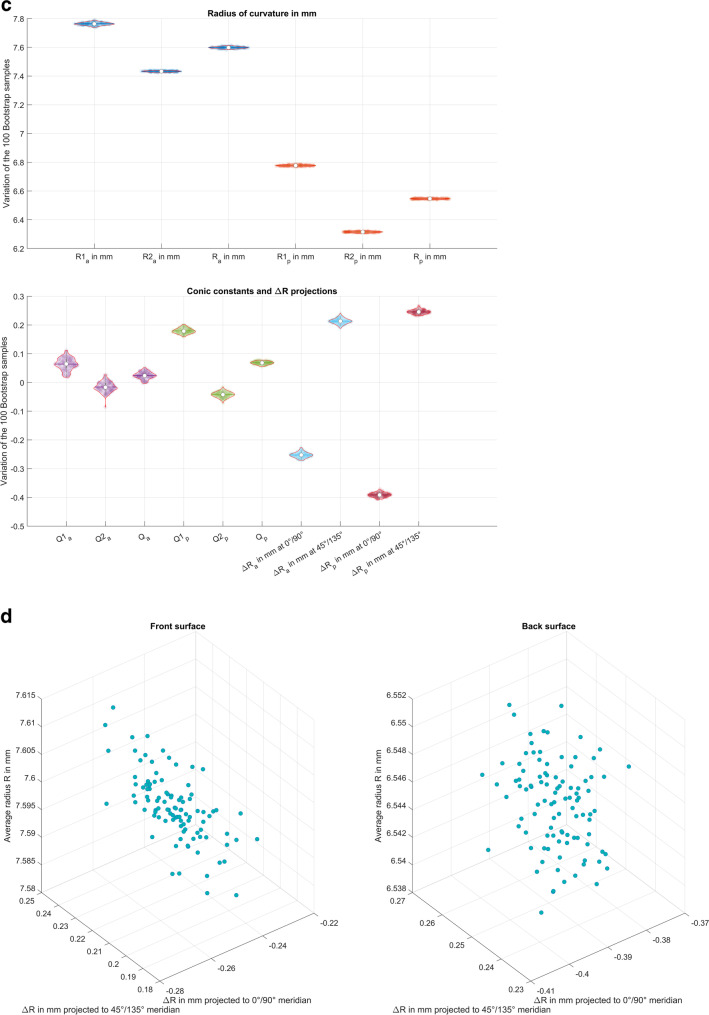
Exemplary case presentation (measurement 11 of *N* = 8000). **a** The fit error for the corneal front (left image) and back surface (right image) derived from the initial Fringe Zernike polynomials decomposition after alignment of the data (removal of decentration and tilt) as a function of the radial component *r* together with the polynomial fit *y*~1+*r*^2^ (polynomial coefficients ·e−4: *a*_0_ = 1.01/8.37, *a*_2_ = 6.08/7.22 for the front/back surface). The fit error is normalised to the polynomial representation before bootstrapping. **b** The Fringe Zernike polynomial representation for the corneal front (left image) and back surface (right image) of the surface evaluated in both cardinal meridian (the flat and the steep meridian) together with a polynomial fit to derive central radius of curvature and asphericity in both meridians. **c** The violin plot of the distributions of the corneal radii of both surfaces (radius in the flat (*R*1) and steep meridian (*R*2), average radius (*R*), and difference of radii in both meridians (Δ*R*)), the corneal asphericities of both surfaces (asphericity in the flat (*Q*1) and steep meridian (*Q*2), average asphericity (*Q*), and difference of asphericities in both meridians (Δ*Q*)), as well as the projections of the difference of both radii to the 0°/90° and the 45°/135 meridian for the corneal front and back surface derived from the *N*_*B*_ = 100 bootstraps of each data. **d** The 3D scatterplot of the average corneal radius (vertical direction) versus the double angle representation of the difference of the radii in both cardinal meridians (horizontal directions) for the corneal front (left image) and the corneal back surface (right image)

To illustrate the principle of our strategy, we selected one case (measurement 11 of 8000) from our dataset as an example. Figure 2a shows the absolute value of the fit error (initial Fringe Zernike decomposition) in the pre-processing stage after alignment of both surfaces (centring in *X* and *Y* and removal of tiles around *X* and *Y*) as a function of the distance to the centre *r* for the corneal front and back surface. As shown in the graph, the fit error *E* increases in a quadratic fashion. The polynomial fit *y*~1+*r*^2^ is used to normalise the fit error *E* to *E*_0_ before bootstrapping. The reconstructed surface FZS in the pre-processing stage is evaluated to obtain both cardinal meridians (the flat (*A*1) and the steep (*A*2) meridian). Figure 2b shows the FZS in both meridians (at axes *A*1 and *A*2) together with the polynomial fit to extract the characteristic surface parameters (the radii and asphericities in cardinal meridians, the average radius and asphericity, and the difference of radii and asphericities between both meridians). After bootstrapping of the normalised fit error from the pre-processing stage, we reversed the normalisation to read out *E*_0_’. Then, for each of the *N*_*B*_ = 100 bootstraps, *E*_0_’ is added to the surface reconstruction FZS, and *N*_*B*_ = 100 Fringe Zernike polynomial decompositions are calculated. Figure 2c displays the distributions of the radii and asphericities in meridians, the average and difference of radii and asphericities between both meridians, and the projections of the difference of radii to the 0°/90° and to the 45°/135° meridian for the corneal front and back surfaces. Finally, Fig. 2d shows the uncertainty of the reconstruction of the radii in terms of average radius and the radius difference between the flat and steep meridian projected to the 0°/90° and to the 45°/135° meridian in a 3D scatterplot for the corneal front and back surface.

Overall, for the 6953 data samples which were analysed in the study, the coefficients for the polynomial fit to normalise the fit error *E* in the radial direction are displayed in Table 1. We can see that the intercept *a*_0_ is mostly systematically larger for the corneal back surface as compared to the front surface, implying either that the measurement data for the corneal back surface are much more noisy or that the data are poorly represented by our Fringe Zernike representation of degree *N*_*Z*_ = 10. In Table 2 and Table 3, the explorative data for the lower and the upper limits of the 90% confidence intervals of the initial 9 Fringe Zernike coefficients derived from the *N*_*B*_ = 100 bootstraps are listed for the corneal front and back surface. These tables show the uncertainties of the Zernike coefficients when the bootstrapped fit errors are reversed in normalisation, added to the initial surface reconstruction FZS, and again decomposed into Fringe Zernike polynomials of degree *N*_*Z*_ = 10. In the lower parts of the tables, the width of the confidence intervals (as upper minus lower boundary of the 90% confidence intervals) is shown. The variations in the Zernike coefficients themselves cannot be directly interpreted in clinical terms. We therefore used the *N*_*B*_ = 100 bootstraps to extract the characteristic parameters for the corneal front and back surface in terms of radii at the flat and the steep meridians, the average radius and difference of radii between the flat and the steep meridians, the asphericities at the flat and the steep meridians, and the average asphericity and difference of asphericities between the flat and the steep meridians, as listed in Table 4. In this table, the median value of the characteristic parameters from the *N*_*B*_ = 100 bootstraps is evaluated, providing a ‘robust’ metric for the surface representation for both corneal surfaces. What we can see from these data is that on average, the radii and the asphericities pretty much resemble the data of classical schematic model eyes. In our population, we found a mean corneal radius of *R*_*a*_ = 7.68/*R*_*p*_ = 6.45 mm and mean asphericities of *Q*_*a*_ = −0.22/*Q*_*p*_ = −0.27 for the corneal front/back surface. In addition, we derived a radius difference between the flat and steep corneal meridian of Δ*R*_*a*_ = 0.13/Δ*R*_*p*_ = 0.17 mm.

**Table 1:** Explorative data of polynomial coefficients for normalisation of the fit error with *E* = *a*_0_+*a*_2_·*R*^2^. The fit error is assumed to increase in a quadratic fashion with the distance *R* from the axis. Data are given with mean, standard deviation (SD), median, and the lower (quantile 5%) and upper (quantile 95%) boundaries of the 90% confidence interval

*N*=6953, data X100	Corneal front surface		Corneal back surface	
	Intercept *a*_0_	2nd-order coefficient *a*_2_	Intercept *a*_0_	2nd-order coefficient *a*_2_
Mean	0.0361	0.0261	0.2572	0.0303
SD	0.0593	0.0322	0.4383	0.0593
Median	0.0209	0.0173	0.1465	0.0148
Quantile 5%	0.0100	0.0092	0.0649	0.0031
Quantile 95%	0.1877	0.1024	1.1941	0.1672

**Table 2:** Explorative data of the lower and upper boundary of the 90% confidence interval and the interquantile range (upper – lower boundary of the 90% confidence interval) of the *N*_*B*_ = 100 bootstraps for the first 9 of 36 Fringe Zernike coefficients for the corneal front surface fit. *Z*1 refers to piston, *Z*2 and *Z*3 to tilt, *Z*4 to defocus, *Z*5 and *Z*6 to the primary astigmatism, *Z*7 and *Z*8 to primary coma, and *Z*9 to spherical aberration

*N*=6953		*Z*1	*Z*2	*Z*3	*Z*4	*Z*5	*Z*6	*Z*7	*Z*8	*Z*9
Lower boundary of the 90% confidence interval over *N*_*B*_ bootstraps	Mean	0.4189	−0.0019	−0.0082	0.4221	−0.0019	0.0056	−0.0026	−0.0115	0.0044
	SD	0.0465	0.0123	0.0174	0.0519	0.0223	0.0278	0.0164	0.0236	0.0165
	Median	0.4123	−0.0012	−0.0033	0.4170	−0.0012	0.0047	−0.0018	−0.0049	0.0059
	Quantile 5%	0.3529	−0.0294	−0.0500	0.3418	−0.0492	−0.0538	−0.0385	−0.0688	−0.0301
	Quantile 95%	0.5303	0.0212	0.0124	0.5381	0.0430	0.0644	0.0297	0.0196	0.0309
Upper boundary of the 90% confidence interval over *N*_*B*_ bootstraps	Mean	0.4242	0.0012	−0.0049	0.4308	0.0009	0.0084	0.0020	−0.0067	0.0100
	SD	0.0469	0.0123	0.0186	0.0522	0.0226	0.0282	0.0165	0.0231	0.0160
	Median	0.4163	0.0008	−0.0012	0.4231	0.0007	0.0067	0.0013	−0.0013	0.0094
	Quantile 5%	0.3653	−0.0217	−0.0445	0.3644	−0.0454	−0.0497	−0.0292	−0.0610	−0.0181
	Quantile 95%	0.5412	0.0282	0.0193	0.5577	0.0501	0.0709	0.0403	0.0296	0.0443
Interquantile range (upper – lower boundary) of the 90% confidence interval over *N*_*B*_ bootstraps	Mean	0.0052	0.0032	0.0033	0.0086	0.0028	0.0029	0.0046	0.0048	0.0056
	SD	0.0092	0.0059	0.0067	0.0154	0.0053	0.0053	0.0083	0.0053	0.0101
	Median	0.0032	0.0019	0.0020	0.0053	0.0017	0.0017	0.0028	0.0029	0.0034
	Quantile 5%	0.0018	0.0011	0.0011	0.0030	0.0010	0.0010	0.0016	0.0016	0.0020
	Quantile 95%	0.0213	0.0132	0.0140	0.0353	0.0118	0.0116	0.0190	0.0203	0.0227

**Table 3:** Explorative data of the lower and upper boundary of the 90% confidence interval and the interquantile range (upper – lower boundary of the 90% confidence interval) of the *N*_*B*_ = 100 bootstraps for the first 9 of 36 Fringe Zernike coefficients for the corneal back surface fit. *Z*1 refers to piston, *Z*2 and *Z*3 to tilt, *Z*4 to defocus, *Z*5 and *Z*6 to the primary astigmatism, *Z*7 and *Z*8 to primary coma, and *Z*9 to spherical aberration

*N*=6953		*Z*1	*Z*2	*Z*3	*Z*4	*Z*5	*Z*6	*Z*7	*Z*8	*Z*9
Lower boundary of the 90% confidence interval over *N*_*B*_ bootstraps	Mean	1.0476	−0.0047	−0.0149	0.0539	−0.0032	0.0184	−0.0067	−0.0215	0.0039
	SD	0.0967	0.0461	0.0382	0.0978	0.0396	0.0416	0.0496	0.0513	0.0439
	Median	1.0349	−0.0019	−0.0059	0.5073	−0.0009	0.0206	−0.0029	−0.0077	0.0101
	Quantile 5%	0.8981	−0.0635	−0.0905	0.3596	−0.0802	−0.0730	−0.0902	−0.1274	−0.0789
	Quantile 95%	1.2943	0.0442	0.0266	0.7082	0.0646	0.0918	0.0606	0.0332	0.0554
Upper boundary of the 90% confidence interval over *N*_*B*_ bootstraps	Mean	1.0619	0.0042	−0.0059	0.5372	0.0046	0.0260	0.0062	−0.0086	0.0183
	SD	0.0167	0.0361	0.0385	0.1030	0.0419	0.0425	0.0479	0.0508	0.0430
	Median	1.0442	0.0019	−0.0012	0.5202	0.0029	0.0252	0.0029	−0.0010	0.0171
	Quantile 5%	0.9114	−0.0436	−0.0741	0.4071	−0.0651	−0.0572	−0.0608	−0.1040	−0.0485
	Quantile 95%	1.3301	0.0635	0.0480	0.7591	0.0793	0.1085	0.0862	0.0630	0.0891
Interquantile range (upper – lower boundary) of the 90% confidence interval over *N*_*B*_ bootstraps	Mean	0.0143	0.0091	0.0091	0.0233	0.0078	0.0076	0.0129	0.0130	0.0144
	SD	0.0616	0.0525	0.0450	0.0968	0.0417	0.0324	0.0721	0.0622	0.0586
	Median	0.0068	0.0037	0.0038	0.0110	0.0033	0.0034	0.0054	0.0055	0.0067
	Quantile 5%	0.0034	0.0019	0.0018	0.0055	0.0016	0.0016	0.0027	0.0027	0.0033
	Quantile 95%	0.0554	0.0396	0.0400	0.0920	0.0326	0.0321	0.0549	0.0558	0.0570

**Table 4:** Explorative data of the median of the characteristic surface parameters derived from the *N*_*B*_ = 100 bootstraps. *R*1 and *R*2 refer to radius of curvature of the flat and steep corneal meridian, *R* to the average radius, and Δ*R* to the difference between flat and steep meridian. *Q*1 and *Q*2 refer to asphericity at the flat and steep corneal meridian, *Q* to the average asphericity, and Δ*Q* to the difference between asphericities at the flat and steep meridian. The table lists the mean, standard deviation (SD), median, and the lower (quantile 5%) and upper (quantile 95%) boundaries of the 90% confidence interval

Median of characteristic parameters of the corneal surface fit derived from *N*_*B*_ = 100 bootstraps									
*N* = 6953		*R*1 in mm	*R*2 in mm	*R* in mm	Δ*R* in mm	*Q*1	*Q*2	*Q*	Δ*Q*
Corneal front surface	Mean	7.7361	7.6180	7.6770	0.1289	−0.1996	−0.2381	−0.2187	0.0385
	SD	0.2275	0.2118	0.2074	0.1362	0.2377	0.2196	0.1929	0.2457
	Median	7.7358	7.6188	7.6841	0.0829	−0.2125	−0.2318	−0.2197	0.0117
	Quantile 5%	7.2454	7.2069	7.2258	0.0047	−0.6797	−0.6562	−0.6099	−0.4078
	Quantile 95%	8.2115	8.0542	8.0802	0.5260	0.3620	0.2805	0.2207	0.6387
Corneal back surface	Mean	6.5190	6.3718	6.4455	0.1683	−0.2262	−0.3093	−0.2676	0.0831
	SD	0.2674	0.2424	0.2416	0.1424	0.2740	0.2492	0.2210	0.2799
	Median	6.5542	6.3816	6.4748	0.1322	−0.2369	−0.3043	−0.2610	0.0551
	Quantile 5%	5.9317	5.9085	5.9243	0.0080	−0.7466	−0.7358	−0.7119	−0.4849
	Quantile 95%	6.9878	6.8744	6.8936	0.5737	0.3498	0.2419	0.2032	0.8056

Table 5 shows the explorative data for the uncertainties (in terms of standard deviations SD) of the characteristic surface data of the *N*_*B*_ = 100 bootstraps for both corneal surfaces, which is a metric for the technical variability of the surface fit and surface representation using OCT measurement data from the Casia2 corneal tomographer. As we can see from the table, the variations in the corneal radii (1.8 μm/3.6 μm) and in the corneal asphericities (0.0054/0.0078) for the corneal front/back surface are on average very low. However, from the 95% quantile, we learn that in 5% of the corneal measurement data, the *N*_*B*_ = 100 bootstraps show a variation of average radius of more than 6.9 μm/15.6 μm and a variation of average asphericity more than 0.022/0.037 for the corneal front and back surface respectively.

**Table 5:** Explorative data of the standard deviation SD of the characteristic surface parameters derived from the *N*_*B*_ = 100 bootstraps as a measure for robustness. *R*1 and *R*2 refer to radius of curvature of the flat and steep corneal meridian, *R* to the average radius, and Δ*R* to the difference between flat and steep meridian. *Q*1 and *Q*2 refer to asphericity at the flat and steep corneal meridian, *Q* to the average asphericity, and Δ*Q* to the difference between asphericities at the flat and steep meridian. The table lists the mean, standard deviation (SD), median, and the lower (quantile 5%) and upper (quantile 95%) boundaries of the 90% confidence interval

Standard deviation SD of characteristic parameters of the corneal surface fit derived from *N*_*B*_ = 100 bootstraps									
*N* = 6953		*R*1 in mm	*R*2 in mm	*R* in mm	Δ*R* in mm	*Q*1	*Q*2	*Q*	Δ*Q*
Corneal front surface	Mean	0.0032	0.0028	0.0018	0.0046	0.0093	0.0082	0.0054	0.0144
	SD	0.0076	0.0079	0.0037	0.0088	0.0182	0.0179	0.0106	0..0289
	Median	0.0019	0.0017	0.0012	0.0027	0.0059	0.0053	0.0034	0.0091
	Quantile 5%	0.0005	0.0002	0.0003	0.0006	0.0019	0.0014	0.0013	0.0034
	Quantile 95%	0.0126	0.0115	0.0069	0.0193	0.0366	0.0312	0.0216	0.0570
Corneal back surface	Mean	0.0059	0.0054	0.0036	0.0081	0.0134	0.0123	0.0078	0.0215
	SD	0.0134	0.0154	0.0092	0.0157	0.0249	0.0262	0.0151	0.0408
	Median	0.0028	0.0024	0.0018	0.0037	0.0070	0.0062	0.0041	0.0109
	Quantile 5%	0.0005	0.0003	0.0003	0.0006	0.0012	0.0009	0.0008	0.0021
	Quantile 95%	0.0302	0.0285	0.0156	0.0432	0.0698	0.0596	0.0371	0.1106

## Discussion

Bootstrapping refers to any test or metrics that uses random sampling with replacement (e.g. simulating a sampling process), which falls under the general envelope of resampling techniques. Bootstrapping is commonly used in computer technology and statistics [12–14], but less well known in ophthalmology (Iskander 2004, [15]). Normally, a set of measurements is performed on the same individuals from the same (intra-operator) or different examiners (inter-operator) to determine how consistent the results are [4]. However, repeat measurements are very time consuming under clinical conditions, and in such cases, bootstrapping offers a practical and valid alternative to repeated measurements for obtaining some insight into the technical variability of measurements. As another alternative, resampling of the data could be used as a straightforward strategy to calculate error propagation.

The idea behind bootstrapping is very simple: Instead of repeating the measurement, the datapoints of a measurement are sampled with replacement to get *N*_*B*_ sets of datapoints of the same size [11]. These *N*_*B*_ sets are considered as ‘repeat measurements’. Model parameters are retrieved from each of the *N*_*B*_ sets and the estimation of the variation of the model parameters is used as a measure of variability of the model fit on the data set. For corneal tomographic data, this means that, e.g. the 4800 data points for the corneal front surface measurement within the 6-mm central zone of the cornea are sampled with replacement *N*_*B*_ = 100 times, and for each of the 100 bootstraps, a surface model is fitted [10]. From the 100 surface models, we can determine the uncertainties of the surface fit, and an explorative analysis gives us metrics (for example, an estimate of how reliable the axes of both cardinal meridians or the radius of curvature or asphericity in both cardinal meridians could be extracted [16]).

In the present study, we used Fringe Zernike polynomials of degree *N*_*Z*_ = 10 (in total 36 components) to represent the surface shape [5]. With a higher degree, the surface model is more flexible to consider surface irregularities, but on the other hand, a higher polynomial degree bears the risk of overfitting, which would mean that measurement noise could be interpreted as measurement data [7, 8, 17]. In general, nearly arbitrary surface models could be fitted to tomographic data, but Zernike polynomials have the advantage of being additive with the consequence that we could derive the coefficients from the 3D data efficiently using simple matrix calculus in terms of a least squares fit [1, 3].

In Fig. 1, the flowchart of our calculation strategy is shown. After alignment of the data, an initial Fringe Zernike polynomial model is fitted to the tomographic data for both surfaces. From the raw measurement data and the surface reconstruction, we extract the fit error, which is used for bootstrapping [18]. As it is well-known that the measurement error increases from the centre to periphery, the fit error had to be normalised before bootstrapping. For normalisation, we used a simple 2nd-order polynomial in the radial direction. From Table 1, we can see that the intercept *a*_0_ is much higher for the back surface as compared to the front surface, meaning that the measurement noise is in general higher for the back surface or that the back surface is not properly described by the surface model. We feel that the Fringe Zernike polynomials with 36 components should be sufficient to represent both corneal surfaces [7], and we therefore argue that the larger intercept *a*_0_ is mostly due to measurement noise. We then analysed the confidence intervals of the Zernike coefficients for the *N*_*B*_ = 100 bootstraps, to obtain insight into the variations resulting from sampling with replacement. In Tables 2 and 3, we restricted the explorative analysis to the lower and upper bounds of the 90% confidence intervals for the first 9 Fringe Zernike coefficients, together with the interquantile range. The characteristic parameters familiar to clinicians were extracted from the Fringe Zernike surface representations. The axes of the cardinal meridians were extracted from the 2 primary astigmatism components *Z*2 and *Z*3, and the Zernike representation was used to calculate the radii and asphericities at both cardinal meridians using a 4th-order polynomial fit. Table 4 lists the median values of the characteristic surface parameters for the 100 bootstraps, and we see that the results pretty much reflect the data of modern schematic model eyes with aspheric surfaces [19]. To account for the robustness of the surface fit, we analysed the standard deviations of the characteristic surface parameters for the 100 bootstraps and these are listed in Table 5. Surprisingly, the radii of curvature (for both meridians as well as the average radius) show very low variation over the 100 bootstraps. In contrast, even though the variation of the asphericity values is in the same range, the relative variation (variation referenced to the mean value as shown in Table 4) is much higher. In general, the variation of the surface fit for the corneal front surface is systematically lower compared to the variation of the surface fit for the corneal back surface, suggesting that the surface fit for the front surface is more robust compared to the back surface.

For a better understanding, we selected one example measurement out of our dataset to illustrate in more detail what we did in this study. In Fig. 2a, we addressed the behaviour of the fit error as the difference between the raw measurement height data and the Fringe Zernike surface reconstruction FZS in radial direction. As we can see, the fit error increases systematically with the distance from the measurement axis, requiring normalisation before bootstrapping (Iskander et al. 2014). The simple polynomial fit *y* = *a*_0_+*a*_2_·*R*^2^ used for normalisation as well as for reversing the normalisation after bootstrapping the error is shown in the graph. The radii and asphericities in both meridians are extracted from an equidistant dense sampling of the Fringe Zernike surface representation, evaluated in the two cardinal meridians as shown in Fig. 2b. Figure 2c displays the distributions of the radii and asphericities in both cardinal meridians (including the average radius and asphericity and the difference of radii and asphericities of the flat and the steep meridian) for the *N*_*B*_ = 100 bootstraps. As we can see from the upper graph, the variation of the radii for both surfaces is very low. However, from the lower graph, we see that the (relative) variation of the asphericities as well as the projections of the radius differences Δ*R* to the 0°/90° and the 45°/135° meridian are systematically larger, implying that the extraction of asphericity from Casia2 data using a Fringe Zernike surface model is less robust compared to the extraction of the overall radius for the corneal front and back surface.

However, the present study has some limitations: firstly, the bootstrapping technique used in this study is based on splitting the surface height data into the surface reconstruction (FZS) and the fit error *E*. The fit error is bootstrapped after normalisation and added after reversing the normalisation again to the FZS. This procedure, although commonly used in bootstrapping, does not necessarily provide a good estimate for the technical variability of the surface fit that would be obtained if repeat measurements were used instead, and the biological variability not addressed. Secondly, for surface representation, we used a Fringe Zernike representation, of radial degree *N*_*Z*_ = 10. The results might be different if other surface models such as quadric surfaces or biconic surfaces were used. Thirdly, due to the large calculation times, we restricted the bootstrap sample size to *N*_*B*_ = 100. A larger sample size could help to derive more reliable results for the confidence intervals of the parameter variability. And fourthly, we used a large dataset of measurements with the Casia2 tomographer from a cataractous population, which might not be representative for measurements of normal subjects.

In conclusion, this study addresses the technical variability of a surface fit for the corneal front and back surface measurement data performed with the Casia2 anterior segment tomographer. In this study, we have shown that bootstrap techniques could be used to estimate the variability of relevant surface parameters such as radii of curvature or asphericity in the cardinal meridians, or Fringe Zernike coefficients from the front and back surface measurement data of an anterior segment optical coherence tomographer. However, comparative studies in the future with a large number of bootstrap samples would be needed to validate whether bootstrapping of single examinations yields equivalent results for uncertainties of characteristic surface parameters as compared to repeat measurements. Based on the uncertainties of the model fit parameters, it would be possible to predict the effect on imaging performance of the eye using raytracing and an error propagation model.

## Declarations

All procedures performed in studies involving human participants were in accordance with the ethical standards of the Ärztekammer des Saarlandes and with the 1964 Helsinki declaration and its later amendments or comparable ethical standards. The local ethics committee has provided a waiver for this study (Ärztekammer des Saarlandes, 157/21). All data processed in this study were already anonymised at the source before being transferred to us for processing. This precludes any back-tracing of the identity, and therefore informed consent of the patients was not necessary.
